# Life in Modern Asylums

**Published:** 1889-04-20

**Authors:** 


### Life in Modern Asylums.—I.

Asylum physicians are frequently asked how they cure their patients, and how they secure to the incurable a certain measure of safety and tranquility. The enquirers seldom seem satisfied with the usual, and truthful reply, that, next to the natural tendency to recovery from some forms of insanity, it is the general system of the management—the daily life led by the patients in the asylum—that is the leading means both of care and of cure. A great deal of misconception still lingers in the popular mind as to the condition of matters within the walls of a modern asylum. The most intelligent and well-informed of the general public, on visiting the wards of an asylum for the first time, express pleased surprise at finding the rooms and their occupants so much more comfortable and tranquil than they had expected. The exaggerated pictures of abuses, which might have been possible under the old systems, delineated in works of fiction of some years back, still linger in the minds of those who have had no opportunity of correcting the false impressions they convey.

So far from the life in asylums being the nightmare of horrors picturesquely daubed by sensational novelists, we repeat that the life in the asylum, the regular daily mode of existence, is by far the most powerful curative agency. Judicious medication will do much to restore the healthy action of the bodily functions, and thus contribute in an important way to the removal of mental disorder; but there are no potions that, acting directly on the spiritual being, can thus cure an attack of insanity.

It is a common error to suppose that all cases of insanity are cases of the same disease. The extraordinarily complex structure of the organ of mind—the brain—and the great delicacy of its functions, render it liable to changes and disorders more numerous and more widely varied than those of any other organ of the body. The treatment in asylums, therefore, to be effective must vary in different cases, and the system of management and discipline must possess a corresponding elasticity.

To give an idea of asylum life to the readers of *The Hospital*, let us trace the beginnings of the disorder in a typical case, accompany the patient to the asylum, and note the treatment he receives there, and the rationale thereof.

Let us suppose our patient in such a social position that the asylum to which he would be sent is one of our County District Asylums.

Let us suppose a person in the prime of life, the father or the mother of a family, with work to do, and responsibilities to bear. Let us suppose that these duties have been satisfactorily performed up to the time when the mental disturbance has caused a failure to fulfil them, with very possibly a painful sense on the part of the patient that things are not right. Let us conceive matters going on from bad to worse, until some force has to be used to restrain the unfortunate individual from unreasonable proceedings, or from actual violence to himself and to those around him. Let us imagine him as still dimly conscious of the burden of his social and domestic duties, and vainly struggling with the new and strange perplexities of his disordered thoughts. Let us picture to ourselves the domestic storms, the father or mother insanely struggling with their children to retain the authority they have become unfitted to wield; let us remember the bewilderment of the relatives, and the ineffective interference of well-meaning but inexperienced friends. Further, let us suppose these painful scenes and struggles to grow rapidly more frequent and severe, and to culminate, it may be, in some alarming and dangerous act of violence or despair.

Insanity is, fortunately, not so common but that many households escape any such visitation, but we have drawn no exaggerated picture of what frequently occurs.

Where there has been violence, the propriety of an immediate removal to an asylum cannot be questioned, unless in the case of persons of great wealth, whose means permit of a private dwelling being converted for the uses of their safe and satisfactory treatment into what is practically a fully equipped asylum. Asylum treatment is necessary in such a case: (1) For the patient's safety; (2) for the safety of others. There are many cases, too, where bodily safety is not directly threatened, in which removal from home becomes absolutely necessary—such as when business matters are grossly mismanaged, or propriety is outraged, or vexatious interference with neighbours has occurred, or where domestic and social ties are strained to the point of rupture.

Besides these paramount considerations of safety to life and property and the integrity of social relationships, removal to an asylum is, in almost all cases, beneficial to the progress of the disease, by freeing the patient from the scenes associated with the beginnings of his disorder, from the pressure of his duties, and from the misdirected efforts of friends—inexperienced as these generally must be, and officiously solicitous in their ministrations. It thus appears that removal to an asylum has, to begin with, a variety of negative advantages.

Allusion must be made, however, to two leading disadvantages of asylum treatment. First, the loss of liberty, though necessary and desirable in his own interests, is real, and is sometimes painfully felt by the patient himself. Much has been done to remove all unnecessary restraint and restriction in asylums in recent years; but the fact may not be overlooked that a certified lunatic is, generally without his own consent, placed under the control of others, and that, though he is exceptionally well looked after by the State, he loses for the time many of the privileges of a citizen. A second great disadvantage is the stigma that, to some extent necessarily, but to a great extent unnecessarily and most unjustly, is attached by society to those who have once lost the use of their reason which loss of reason is indelibly stamped on the record of their lives by the very proceedings which the law, jealous of personal liberty, has made essential for legal confinement. The name appears in official documents and registers, and from these it cannot be rubbed out. It is true, and becoming more and more generally admitted, that sympathy should be accorded and no blame attached to one who labours under this misfortune; but the common sense of mankind does not, and should not, ignore the warning of a previous attack, nor disregard the possible consequences of a return of the mental disorder in the patient or his descendants in any dealings he may have with them. A man is not made more or less insane by removal to an asylum, but his registration as a lunatic definitely marks him as one who has passed beyond the border land.

We have now brought our patient to the threshold of the asylum where it has been determined to send him. How he fares therein on admission we shall attempt to describe in a future paper.

(*To be continued.*)

---

"Scoff not at the dreamer who expects in the material world a revolution similar to that which has already taken place in the domains of thought. The thought goes before the deed, as the lightning precedes the thunder."—*Heine.*

